# Loss of Mob1a/b impairs the differentiation of mouse embryonic stem cells into the three germ layer lineages

**DOI:** 10.1038/s12276-019-0342-z

**Published:** 2019-11-14

**Authors:** June Sung Bae, Sun Mi Kim, Yoon Jeon, Juyeon Sim, Ji Yun Jang, Jaehyung Son, Woosol Hong, Mi Kyung Park, Ho Lee

**Affiliations:** 1 Research Institute, National Cancer Center, Goyang, Gyeonggi 10408 Republic of Korea; 2 Graduate School of Cancer Science and Policy, National Cancer Center, Goyang, Gyeonggi 10408 Republic of Korea

**Keywords:** Genetic engineering, Regenerative medicine, Embryonic stem cells

## Abstract

The Hippo pathway plays a crucial role in cell proliferation and apoptosis and can regulate stem cell maintenance and embryonic development. MOB kinase activators 1A and 1B (Mob1a/b) are key components of the Hippo pathway, whose homozygous deletion in mice causes early embryonic lethality at the preimplantation stage. To investigate the role of Mob1a/b in stem cell maintenance and differentiation, an embryonic stem cell (ESC) clone in which Mob1a/b could be conditionally depleted was generated and characterized. Although Mob1a/b depletion did not affect the stemness or proliferation of mouse ESCs, this depletion caused defects in differentiation into the three germ layers. Yap knockdown rescued the in vitro and in vivo defects in differentiation caused by Mob1a/b depletion, suggesting that differentiation defects caused by Mob1a/b depletion were Yap-dependent. In teratoma experiments, Yap knockdown in Mob1a/b-depleted ESCs partially restored defects in differentiation, indicating that hyperactivation of Taz, another effector of the Hippo pathway, inhibited differentiation into the three germ layers. Taken together, these results suggest that Mob1a/b or Hippo signaling plays a critical role in the differentiation of mouse ESCs into the three germ layers, which is dependent on Yap. These close relationship of the Hippo pathway with the differentiation of stem cells supports its potential as a therapeutic target in regenerative medicine.

## Regenerative medicine: Possible pathway to improve therapies

Insights into how embryonic stem cells differentiate in the earliest stages of growth reveal a potential target for regenerative therapies. The Hippo pathway and its key factors, Mob1a and Mob1b, play crucial roles in stem cell maintenance and embryonic development, but their mechanisms are unclear. To investigate, Ho Lee at the National Cancer Center, South Korea, and co-workers conducted experiments on cloned mouse embryonic stem cells (ESCs). They found that depleting Mob1a/b prevented ESCs from differentiating into the three embryonic layers required for organ and tissue growth. They found that the activity of Yap, the Hippo pathway effctor, is essential for the failure to differentiate in ESCs lacking the factors. The Hippo pathway, and possibly Yap, could provide therapeutic targets, although caution is needed in manipulating Yap because of its association with cancer development.

## Introduction

Embryonic stem cells (ESCs) are derived from the pluripotent inner cell mass (ICM) cells of the blastocyst-stage embryo^1^. Because ESCs are capable of self-renewal and can differentiate into every cell type in the animal body, the properties of pluripotent stem cells have generated much interest for their use as potential therapies for defects in developmental and regenerative processes in human diseases. Recently, researchers have found that pluripotent stem cells and progenitor cells can aggregate and generate tissue structures known as organoids, which resemble primary tissues in vivo^2^. Although the current culture system of organoids does not include endothelial or stromal cells, in vitro organoid models can provide useful tools for basic research and clinical applications^2^.

Transcription factors and chromatin regulators are important for maintaining the pluripotency of ESCs^3^. The pluripotent state of ESCs is largely dependent on the core transcription factors Oct4, Sox2 and Nanog^4–7^. Reprogramming of differentiated adult cells into induced pluripotent stem cells is also possible through the expression of a set of transcription factors, such as Oct4, Sox2, Klf4, and Myc^8^. These core transcription factors activate the expression of genes necessary to maintain ESC properties and the pluripotent state of ESCs. They also contribute to the repression of genes encoding cell-lineage-specific regulators^9–11^. Many additional regulators for maintaining stemness have been found by genetic and proteomic screening^12,13^.

In mammals, Yes-associated protein 1 (Yap) and WW domain-containing transcription regulator protein 1 (Taz) are negatively regulated by the activation of the core components of the Hippo pathway^14^. These Hippo core components are essential during early embryonic development. *Yap*^−/−^:*Taz*^−/−^ mouse embryos showed embryonic lethality before the 16-cell morula stage and lacked TE lineage specification^15^. Depletion of Nf2, Amot, or Lats1/2 led to failure to develop ICM-derived lineages^16–18^. The Hippo pathway has also emerged as a crucial regulator of the stemness of ESCs. The Yap-Tead2 complex can upregulate *Oct4* and *Nanog* expression in mouse ESCs^19^. Furthermore, overexpression of Yap prevents the differentiation of ESCs, and knockdown of Yap leads to the loss of the pluripotency of ESCs^20^. Taz is also required for the translocation of Smad2/3/4 into the nucleus to maintain TGFβ signaling and the pluripotent state of human ESCs^21^. Therefore, the Hippo signaling pathway plays a role in maintaining pluripotency and determining cell fate specification either directly via the control of core transcription factors (e.g., Oct4) or indirectly by mediating other signaling pathways (e.g., SMAD pathway) in ESCs. Additionally, it was reported that increasing Yap activity promoted stemness and inhibited differentiation in many organs and tissues^22^, indicating that the Hippo pathway could be a potential target for organ regeneration and repair upon injury.

MOB1 is a regulator of mitosis in yeast^23–26^. Deletion of the *dMob1* gene triggers tumor development in *Drosophila*, where dMob1 acts as a tumor suppressor^27^. In humans, MOB1A and MOB1B are involved in cell proliferation, cell-lineage specification, and mitotic exit^28–31^. MOB1A/B can also activate NDR/LATS1 kinases^32,33^ and is a core component of the Hippo pathway along with MST1/2, LATS1/2, and SAV1^14^. Phosphorylation of MOB1A/B by the MST1/2 kinase is required for the interaction of MOB1A/B with the NDR/LATS kinases^34–36^. Binding of MOB1A/B to the LATS1/2 kinase fully activates LAST1/2 activity, which in turn phosphorylates YAP and TAZ, inhibiting their activity^37^. Therefore, Mob1a/b is an essential core component of the Hippo pathway that prevents indiscriminate Yap/Taz hyperactivity.

In this study, we generated and characterized a mouse ESC clone in which Mob1a/b could be conditionally depleted to investigate whether the stemness or differentiation potency of ESCs was modulated by Mob1a/b. We found that Mob1a/b could control the differentiation of mouse ESCs into the three germ layers, which was dependent on Yap.

## Materials and methods

### Generation of Mob1a and Mob1b knockout mouse

A 15-kb DNA fragment containing exons of the murine *Mob1a* and *Mob1b* genes was retrieved from BAC clones (bMQ-423L2 and 240C9, respectively) into a pBluescript phagemid system using a previously reported procedure^38^. The generation of targeted ES cell clones and germline transmission of the *Mob1a*^*puro*^ and *Mob1b*^*puro*^ alleles are described in Supplementary Fig. 1. Targeting strategies of *Mob1a*^*flox*^ and *Mob1b*^*flox*^ alleles were performed as described previously^39^ and in Supplementary Fig. 2. All mouse strains were backcrossed for more than six generations to C57BL/6J. This study was reviewed and approved by the Institutional Animal Care and Use Committee of the National Cancer Center Research Institute.

To generate ESC lines, embryos during the blastocyst stage were harvested from the uterus of a pregnant female mouse using M2 medium (Sigma-Aldrich). Individual embryos were transferred to mitomycin C (Sigma-Aldrich)-treated primary mouse embryonic fibroblast (MEF) feeders and cultured in ESC medium, which consisted of high glucose Dulbecco’s modified Eagle’s medium (Welgene, Republic of Korea), 15% serum replacement (Gibco), 2 mM l-glutamine (Gibco), 1% non-essential amino acids solution (Gibco), 0.1% β-mercaptoethanol (Gibco), 5% penicillin–streptomycin (Gibco) and 0.01% recombinant mouse LIF protein (Chemicon). After 7 days, cells were incubated with medium supplemented with 3 μM CHIR99021 (Sigma-Aldrich) and 1 μM PD035901 (Selleckchem) for 1 or 2 weeks. The genotype of each clone was identified following PCR as described in Supplementary Fig. 2.

### Culture and differentiation of mESCs

Undifferentiated mouse ES cells were routinely maintained on a tissue culture plate coated with mitomycin C-treated primary MEF feeder in ESC medium at 37°C in a humidified atmosphere containing 5% CO_2_ as previously described. For depletion of Mob1a/b, *Mob1a*^f/f^:*Mob1b*^f/f^:*CAGGCre-ER*^*TM*^ mouse ESCs were treated for at least 3 days with 0.5 µM 4-hydroxytamoxifen (Sigma-Aldrich) diluted in ethanol.

For differentiation experiments, feeders were depleted by a 30-min incubation on the tissue culture plate, followed by gentle agitation for purifying ESCs. Embryoid bodies (EBs) were generated using the hanging drop method. Cells were incubated (5×10^2^ cells per 35 µl) on the lid of a tissue culture dish in differentiation media. The EBs were maintained in suspension culture for 4 days (2 days as hanging drops and 2 days in bacteriological-grade Petri dishes), and on day 5, EBs were plated on tissue culture plates coated with 0.1% gelatin for attachment and spreading for 2 days.

### Immunoblot analysis

Harvested ESCs were lysed with RIPA buffer (GenDepot) containing Xpert proteinase inhibitor cocktail and phosphatase inhibitors (GenDepot). The protein concentration in each lysate was quantified with a protein assay dye reagent (Bio-Rad). Fifteen micrograms of lysate was fractionated on an 8–13% gradient sodium dodecyl sulfate-polyacrylamide gel and electroblotted on nitrocellulose membranes (Bio-Rad). Blots were incubated with primary antibodies in 0.05% Tween-20/TBS (TBST)-based solution at 4 °C overnight on a shaker and corresponding horse radish peroxidase-conjugated secondary antibodies (GenDepot) at room temperature for 40 min on a shaker. Chemiluminescence detection was performed with the standard protocol. Antibodies are listed in Supplementary Table 1.

### Histology and immunohistochemistry

Teratomas were isolated and fixed at 4 °C overnight with fresh 4% paraformaldehyde in phosphate-buffered saline (PBS) and then embedded in paraffin. Five-micrometer paraffin sections were prepared using a microtome and stained with hematoxylin and eosin. For immunohistochemical staining, the sections were deparaffinized and rehydrated using the standard protocol. Antigen retrieval was performed in a solution (10 mM trisodium citrate, pH 6.0/0.05% Tween-20) by boiling for 10 min in a microwave oven. The tissue sections were incubated with blocking solution (10% goat serum, 1% bovine serum albumin/Tris-buffered saline (BSA/TBS) for 1 h at room temperature and reacted with anti-Taz antibody (Sigma-Aldrich) at 4 °C overnight and corresponding biotinylated secondary antibody diluted 1:500 in 1% BSA/TBS at room temperature for 1 h. The slides were incubated in 0.3% hydrogen peroxide in TBS for 15 min to block endogenous peroxidase. An ABC avidin-biotin-DAB detection kit (Vector Labs, Burlingame, CA, USA) was then used for the detection and visualization of staining according to the supplied protocol. Finally, slides were counterstained with hematoxylin and dehydrated for coverslip mounting. Images were obtained using Observer.Z1 or Imager.M1 (Zeiss).

### RNA isolation, complementary DNA synthesis, and semiquantitative/quantitative PCR

Total RNA was isolated from cells using TRIzol® reagent (Life Technologies) with phase separation by chloroform and ethanol precipitation. The RNA pellet was dissolved in diethyl pyrocarbonate water (500 ng/µl). Total RNA was reverse transcribed using ReverTra Ace® qPCR RT Master Mix (Toyobo). Semiquantitative PCR with reverse transcription (semi-RT-qPCR) was performed using EmeraldAmp GT PCR Master Mix (Takara). RT-qPCR was performed in triplicate for each sample using SYBR® Premix Ex Taq TM II (Takara) on a LightCycler® 480 Real-Time PCR System (Roche)^40^. Sequences of oligonucleotides are listed in Supplementary Table 2. PCR primers for the *Mob1a/b* alleles were designed to identify the chromosomal regions deleted by Cre-*loxP*-mediated recombination.

### Alkaline phosphatase staining

Cells were fixed in −20 °C methanol for 10 min and stained using the Alkaline Phosphatase kit (Vector) according to the manufacturer’s instructions.

### Cell cycle analysis

Cells were fixed overnight in 70% ethanol (EtOH) at 4 °C and washed with PBS and incubated in 0.5 mg/ml RNase A solution (Sigma-Aldrich) at 37 °C for 10 min. Then cells were stained with 50 μg/ml propidium iodide (Sigma-Aldrich) for 30 min^41^. Cell cycle distribution was assessed by a BD FACSCalibur^TM^ (BD Biosciences).

### Lentiviral infection

To generate the Yap-knockdown cells, mouse ESCs were infected with lentivirus containing short hairpin RNA (shRNA) targeting Yap. Lentiviral packaging plasmids pLP1, pLP2, and pLP/VSVG and pLKO.1-Blasticidine or pLKO.1-puromycin construct were cotransfected into 293FT cells using JetPEI (Polyplus-transfection). At 48 h after transfection, viral supernatant was supplemented with 10 µg/ml polybrene, filtered through a 0.45 µm filter, and used to infect mouse ESCs. The next day, the media were changed with fresh ESC medium, and at 48 h after infection, the cells were selected with 20 µg/ml blasticidin and 2 µg/ml puromycin (Sigma-Aldrich). The target sequences used in the knockdown experiments were as follows. shYap, 5′-TGA GAA CAA TGA CAA CCA ATA-3′; shTaz #1, 5′-GAT GAA TCC GTC CTC GGT G-3′; shTaz #2, 5′-CAG CCG AAT CTC GCA ATG AAT-3′; shTaz #3, 5′-CCT GCA TTT CTG TGG CAG ATA-3′; shLats1 #1, 5′-GCC CAA CAG GAA CAG TCA TAA-3′; shLats1 #2, 5′-GCA ACA TTC AAT TAA CCG AAA-3′; shLats1 #3, 5′-CCT ATT CAA CAG CCC GTG AAA-3′; shLats2 #1, 5′-CTC TCA GGG AAA TCC GAT ATT-3′; shLats2 #2, 5′-CGC AAG AAT AGC AGA GAT GAA-3′; shLats2 #3, 5′-CGC CTT CTA TGA GTT CAC CTT-3′.

### Teratoma formation assay

For the teratoma formation assay, mouse ESCs (1.5 × 10^6^ cells) suspended in 50 µl of PBS were mixed with 50 μl of Matrigel (Corning), and then subcutaneously injected into BALB/c nude mice (Orient Bio, Korea). Teratomas were recovered by dissection with surrounding tissue at 6 weeks after injection. Tumors were fixed in 4% paraformaldehyde and embedded in paraffin. Five-micrometer tissue sections were prepared using a microtome and stained with hematoxylin and eosin.

### Statistical analysis

Statistical analysis (unpaired two-tailed Student’s *t* test) was performed using GraphPad Prism 5 software. For all experiments with error bars, data are presented as the mean ± SEM. A value of *p* < 0.05 was considered to be significant; *p *<* 0.05, **p < 0.01, and ***p < 0.001.

## Results

### Depletion of Mob1a/b causes early embryonic lethality in mice

To explore the physiological function of Mob1a/b in embryonic development, we generated conditional-knockout *Mob1a* and *Mob1b* alleles by gene targeting in mouse ESCs. In the gene targeting strategy, exon 2 of the *Mob1a* allele and exon 3 of the *Mob1b* allele were flanked by two *loxP* sequences, which were recognized and removed by Cre recombinase. Consequently, only the first 75 of the 651 nucleotides of *Mob1a* and 216 of the 651 nucleotides of *Mob1b* were correctly transcribed. These aberrant messenger RNA transcripts contained premature stop codons and were degraded by the nonsense-mediated decay pathway (Supplementary Fig. 2). Because single-homozygous mutants (*Mob1a*^−/−^:*Mob1b*^+/+^ or *Mob1a*^+/+^:*Mob1b*^−/−^) were viable and did not show any abnormalities until more than 18 months after birth, we generated the double-homozygous mutant (*Mob1a*^−/−^:*Mob1b*^−/−^). Double-homozygous mutants from heterozygote intercrosses were not viable and showed embryonic lethality before E8.5 (Table 1a, b). These results indicated that Mob1a and Mob1b have mutual redundancy and that one of the two proteins is required for early embryogenesis in mice.

**Table 1:** Depletion of Mob1a/b causes early embryonic lethality in mice: (a) The number and genotypes of pups from *Mob1a*^+/−^:*Mob1b*^+/−^ intercrosses. (b) No *Mob1a/b*-null embryos were observed from *Mob1a*^+/−^:*Mob1b*^−/−^ intercrosses (upper, designated as Mob1b^−/−^ background) or *Mob1a*^−/−^:*Mob1b*^+/−^ intercrosses (bottom, designated as Mob1a^−/−^ background)

(a)			
*Mob1a*	*Mob1b*	No. (%) of mice	
+/+	+/+	4 (2.8)	
+/−	+/+	12 (8.5)	
−/−	+/+	6 (4.2)	
+/+	+/−	19 (12.8)	
+/−	+/−	36 (25.7)	
−/−	+/−	26 (18.5)	
+/+	−/−	10 (7.1)	
+/−	−/−	18 (20.0)	
−/−	−/−	0 (0)	

(b)			
	+/+	+/−	−/−
E8.5d (*Mob1b*^−/−^ background)	4	8	0
E10.5d (*Mob1a*^−/−^ background)	6	10	0

### Depletion of Mob1a/b has little effect on the maintenance of stemness/pluripotency or proliferation of mouse ESCs

**Fig. 1:**
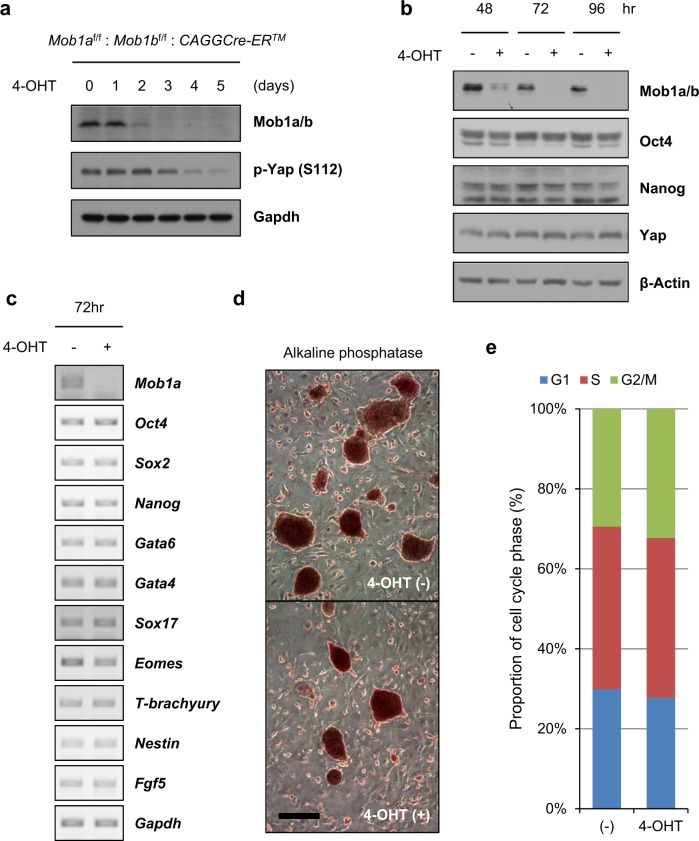
Mob1a/b has little effect on the maintenance of stemness and proliferation in mouse ESCs. **a** Immunoblot analysis for Mob1a/b and p-Yap (S112) in lysates from *Mob1a*^f/−^:*Mob1b*^f/f^:*CAGGCre-ER*^*TM*^ mouse ESCs harvested at the indicated times after 4-OHT treatment. Gapdh served as a loading control. **b** Immunoblot analysis for Mob1a/b, Oct4, Nanog, and Yap in lysates from *Mob1a*^f/−^:*Mob1b*^f/f^:*CAGGCre-ER*^*TM*^ mESCs harvested at the indicated times after 4-OHT treatment. GAPDH served as a loading control. **c** Semiquantitative PCR for *Mob1a*, pluripotency markers (*Oct4*, *Sox2*, and *Nanog*), and differentiation markers (*Gata6*, *Gata4*, *Sox17*, *Eomes*, *T-brachyury*, *Nestin*, and *Fgf5*) in *Mob1a*^f/−^:*Mob1b*^f/f^:*CAGGCre-ER*^*TM*^ ESCs. **d** Representative images of alkaline phosphatase staining of *Mob1a*^f/−^:*Mob1b*^f/f^:*CAGGCre-ER*^*TM*^ ESCs on the seventh day after 4-OHT treatment. Scale bars, 200 μm. **e** Cell cycle analysis of *Mob1a*^f/−^:*Mob1b*^f/f^:*CAGGCre-ER*^*TM*^ mouse ESCs on the seventh day after 4-OHT treatment

To investigate whether the failure of embryogenesis under *Mob1a/b* gene deletion is caused by loss of stemness or aberrant differentiation of stem cells, we generated *Mob1a*^*f/f*^:*Mob1b*^*f/f*^:*CAGGCre-ER*^*TM*^ mouse ESCs in which the *Mob1a/b* genes could be deleted by 4-hydroxytamoxifen (4-OHT) treatment and evaluated the effects of Mob1a/b depletion on markers of pluripotency and differentiation. Mob1a/b proteins were nearly depleted after 3 days of 4-OHT treatment (Fig. 1a). Mob1a/b depletion caused a decrease in Yap-S112 phosphorylation and upregulation of Taz protein levels (Fig. 1a and Supplementary Fig. 3), which confirmed that Lats1/2 kinase activity, Yap phosphorylation, and Taz protein expression are modulated by Mob1a/b in mouse ESCs. The levels of the pluripotency-related markers Oct4 and Nanog in Mob1a/b-depleted ESCs were similar to those of the control (Fig. 1b). The expression levels of differentiation-related markers (*Gata6*, *Gata4*, *Sox17*, *Eomes*, *T-brachyury*, *Nestin*, and *Fgf5*) were also unchanged following Mob1a/b depletion (Fig. 1c).

We next investigated whether the in vitro maintenance of mouse ESCs was influenced by Mob1a/b depletion. Mob1a/b-depleted ESCs were maintained for 1 week, followed by observing the ES cell morphology and visualizing the pluripotency using alkaline phosphatase staining. Consistent with the marker analysis (Fig. 1b), the cell morphology and alkaline phosphatase activity of the Mob1a/b-depleted ESCs were similar to those of the control (Fig. 1d). Mob1a/b depletion also did not affect cell cycle progression (Fig. 1e). These results suggest that Mob1a/b depletion does not affect the maintenance of stemness/pluripotency or proliferation of mouse ESCs.

### Mob1a/b is required for the differentiation of mouse ESCs into the three germ layers

**Fig. 2:**
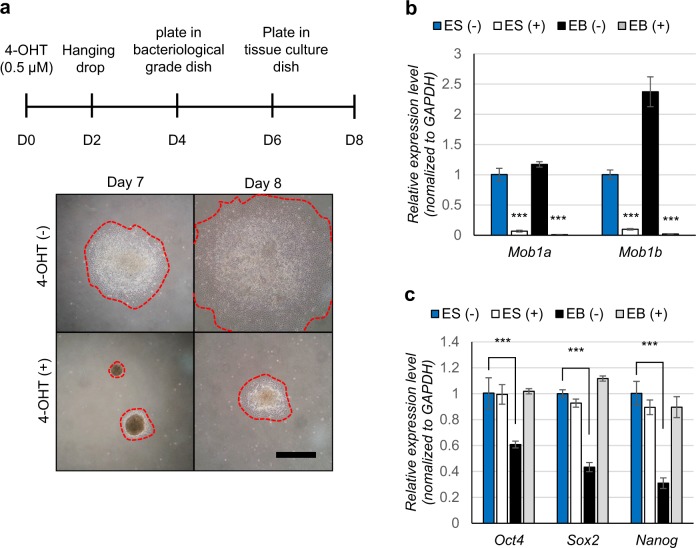
Mob1a/b is essential for the differentiation of mouse ESCs. **a** Schematic diagram of EB formation and representative images of EB outgrowth on tissue culture dishes on days 7 and 8 after EB formation. Dotted lines indicate EB outgrowth. Scale bar, 400 μm **b**, **c** Quantitative PCR for *Mob1a/b* and pluripotency markers (*Oct4*, *Sox2*, and *Nanog*) in mESCs and EBs. ES (−), ESCs without 4-OHT treatment; ES (+), 4-OHT-treated ESCs; EB (−), EB without 4-OHT treatment; EB (+), 4-OHT-treated EB. Data are presented as the mean ± SEM. ***P < 0.001

Because *Mob1a/b* gene deletion showed little effect on the maintenance of stemness and proliferation of mouse ESCs, we investigated whether Mob1a/b depletion affected the differentiation of mouse ESCs. EB formation is typically used as a tool to initiate spontaneous differentiation of ESCs into the three germ layers. The outgrowth of EBs after attachment to a tissue culture dish indicates the expansion of the endodermal-lineage cells^42,43^. In this experiment, we generated EBs using the hanging drop method and then performed the EB migration assay. After plating the EBs on a tissue culture dish coated with 0.1% gelatin, the outgrowth and migration of Mob1a/b-depleted EBs were dramatically reduced compared to the control, suggesting that Mob1a/b-depleted ESCs were defective in the differentiation into the endodermal lineage (Fig. 2a).

**Fig. 3:**
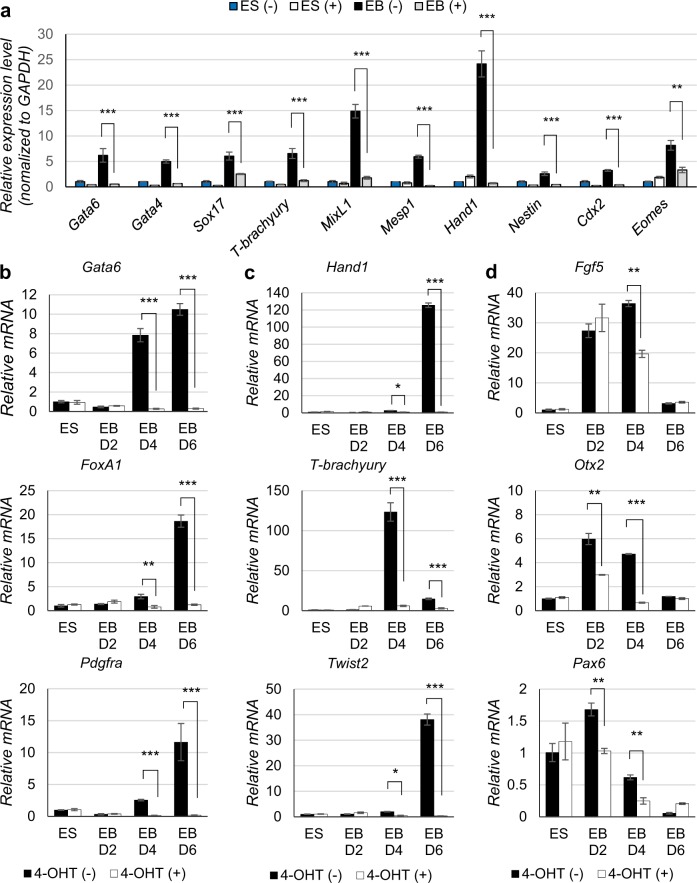
Mob1a/b knockout causes a defect in the differentiation of mouse ESCs. **a** Quantitative PCR for germ layer markers (endoderm: *Gata6*, *Gata4*, *Sox17*; mesoderm: *T-brachyury*, *MixL1*, *Mesp1*, *Hand1*; ectoderm: *Nestin*; trophectoderm: *Cdx2, Eomes*) in mouse ESCs and EBs. **b**–**d** Quantitative PCR for germ layer markers at different stages of EB formation. (**b**, endoderm; **c**, mesoderm; **d**, ectoderm). ES, ESCs before EB formation; EB D2, EB after 2-day culture (hanging drop stage); EB D4, EB after 4-day culture (in bacteriological-grade Petri dish); EB D6, EB after 6-day culture (in cell and tissue culture dish). Data are presented as the mean ± SEM. *P < 0.05, **P < 0.01, ***P < 0.001

To further characterize Mob1a/b-depleted EBs, we performed RT-qPCR for the pluripotency markers *Oct4*, *Sox2*, and *Nanog*. While the expression levels of *Oct4*, *Sox2*, and *Nanog* dramatically decreased by 39.5%, 56.7%, and 69.1%, respectively, in the control EBs under differentiation conditions, they were not changed in the Mob1a/b-depleted EBs (Fig. 2b, c). We also investigated the differentiation status of the Mob1a/b-deficient EBs using RT-qPCR for markers of the three germ layers. The expression of the endoderm lineage markers (*Gata6*, *Gata4*, *Sox17*, *FoxA1*, and *Pdgfra*) increased 10- to 20-fold on days 4 and 6 of EB formation in the control. In contrast, the expression of these markers did not increase in the Mob1a/b-depleted EBs (Fig. 3a, b). The expression levels of the mesoderm lineage markers (*Hand1*, *T-brachyury*, *Twist2*, *Mesp1*, and *MixL1*) increased 40- to 120-fold on days 4 and 6 of EB formation in the control, but were not induced during differentiation of the Mob1a/b-depleted ESCs (Fig. 3a, c). Furthermore, the ectoderm markers (*Fgf5*, *Otx2*, and *Pax6*) in the Mob1a/b-depleted EBs were induced to a lesser extent during differentiation compared to the control (Fig. 3a, d). In the experiments with Lats1/2-knockdown ESCs, we also observed an in vitro defect in the differentiation into the three germ layers (Supplementary Fig. 4). These results suggest that Mob1a/b or Lats1/2 depletion in mouse ESCs causes a defect in the differentiation of ESCs into the early three germ layers in vitro.

### The differentiation defects caused by Mob1a/b depletion are Yap-dependent

Mob1a/b is a scaffold protein that activates the Lats1/2 kinases, which phosphorylate and inactivate the Yap transcriptional activity by nuclear delocalization, cytoplasmic sequestration, and induction of proteasomal degradation^37^. We investigated the levels of Yap protein and S112 phosphorylation upon differentiation of mouse ESCs (Supplementary Fig. 3). Phosphorylation of Yap-S112 increased upon EB formation of wild-type ESCs, indicating that Yap activity was inhibited upon differentiation of mouse ESCs into the three germ layers. The depletion of Mob1a/b caused a decrease in Yap-S112 phosphorylation (i.e., Yap hyperactivation), suggesting that Yap activity or its phosphorylation was modulated by Mob1a/b.

**Fig. 4:**
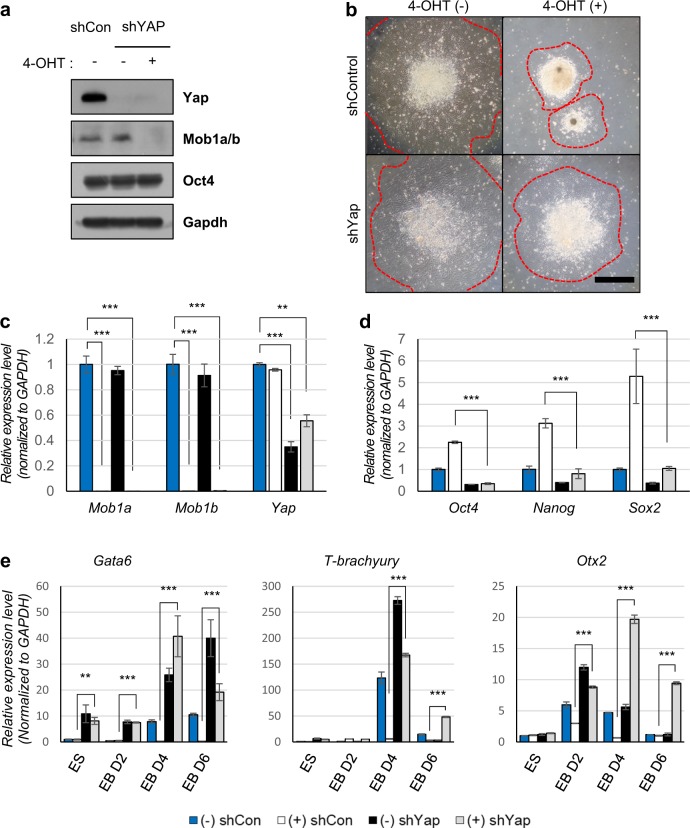
The function of Mob1a/b in the differentiation of mouse ESCs is dependent on Yap activity. **a** Immunoblot analysis for Yap, Mob1a/b, and Oct4 in lysates from *Mob1a*^f/f^:*Mob1b*^f/f^:*CAGGCre-ER*^*TM*^ ESCs after 4-OHT and shYap treatment. GAPDH served as a loading control. **b** Representative images of EB outgrowth on tissue culture dishes eight days after EB formation. Dotted lines indicate EB outgrowth. Scale bar, 100 μm **c**, **d** Quantitative PCR for *Mob1a/b*, *Yap*, and pluripotency markers (*Oct4*, *Sox2*, and *Nanog*) on day 6 after EB formation. **e** Quantitative PCR for germ layer markers (endoderm, *Gata6*; mesoderm, *T-brachyury*; ectoderm, *Otx2*) at the different stages of EB formation. (−) shCon, shControl ESCs without 4-OHT treatment; (+) shCon, 4-OHT-treated shControl ESCs; (−) shYap ESCs without 4-OHT treatment; (+) shYap, 4-OHT-treated shYap ESCs. Data are presented as the mean ± SEM. **P < 0.01, ***P < 0.001

Therefore, we hypothesized that the defects in ESC differentiation caused by Mob1a/b depletion were due to the hyperactivation of Yap, and these defects would be rescued by Yap knockdown. To investigate this hypothesis, we knocked down *Yap* expression using shRNA gene silencing in Mob1a/b-depleted ESCs (Fig. 4a) and then performed EB formation and migration assays to evaluate the differentiation potency of these mouse ESCs.

Until the fourth day of EB formation, Yap knockdown had little effect on the morphology of Mob1a/b-depleted mouse ESCs. On day 6, Yap knockdown increased the outgrowth of the Mob1a/b-depleted EBs (Fig. 4b). These data suggested that Yap knockdown could restore the defects in EB outgrowth caused by Mob1a/b depletion. Next, we analyzed the stem cell and germ layer markers using RT-qPCR in ESCs treated with 4-OHT, shYap, or both at day 6 of EB formation (Fig. 4c). Under differentiation conditions, Yap knockdown reduced the expression levels of *Oct4*, *Sox2*, and *Nanog* and increased the markers of the three germ layers in the Mob1a/b-depleted cells (Fig. 4d, e). These results support the hypothesis that Mob1a/b depletion causes defects in the differentiation of ESCs into the three germ layer lineages in a Yap-dependent manner.

### Mob1a/b is required for the differentiation of mouse ESCs in vivo

**Fig. 5:**
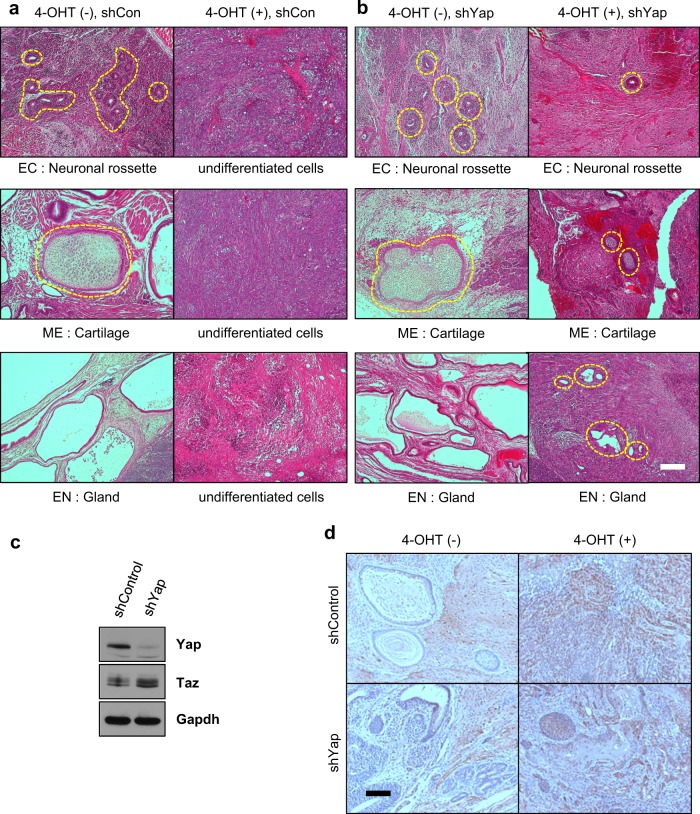
Mob1a/b is required for the differentiation of mouse ESCs in vivo. **a**, **b** Representative images of hematoxylin and eosin staining images of a teratoma originating from *Mob1a*^f/f^:*Mob1b*^f/f^:*CAGGCre-ER*^*TM*^ ESCs. Teratomas were recovered by dissection with the surrounding tissue 6 weeks after mouse ESC injection. Scale bar, 200 μm. **c** Immunoblot analysis for Yap and Taz in lysates of *Mob1a*^f/f^:*Mob1b*^f/f^:*CAGGCre-ER*^*TM*^ ESCs after shYap treatment. Gapdh served as a loading control. **d** Representative images of immunohistochemical staining of a teratoma using an anti-Taz antibody. EC, ectodermal tissue; ME, mesodermal tissue; EN, endodermal tissue. Scale bar, 200 μm

To identify whether Mob1a/b is required for spontaneous differentiation into tissues of the three germ layers *in vivo*, which is Yap-dependent, we performed teratoma formation assays. Teratomas are tumors commonly composed of multiple cell types and tissues derived from more than one germ layer. Teratomas generated with wild-type ESCs were composed of endodermal, mesodermal, and ectodermal tissues. In contrast, teratomas formed from Mob1a/b-depleted ESCs were composed of tissues made up of undifferentiated cells that did not have any of the structural characteristics of the three germ layer cells, indicating that Mob1a/b is required for the differentiation of mouse ESCs into the three germ layers in vivo (Fig. 5a). Furthermore, downregulation of Yap in the Mob1a/b-depleted ESCs resulted in the formation of teratomas comprised of differentiated tissues that were smaller than in the teratomas generated with wild-type ESCs (Fig. 5b). These results suggest that Yap and additional factor(s) are involved in the in vivo defects in the differentiation of Mob1a/b-depleted ESCs into the three germ layers.

The transcriptional coactivator Taz is a closely related paralog of Yap and has functional redundancy with Yap in early embryos and cardiac growth and regeneration^15,44^. It also shares transcriptional targets with Yap^45,46^. Taz was upregulated in Yap-knockdown ESCs (Fig. 5c). In the teratoma formation assay with wild-type ESCs, we found that Taz was primarily localized to undifferentiated cells that did not show any structural characteristics of the three germ layers (Fig. 5d). In the Mob1a/b-depleted teratomas, there was extensive Taz expression in most of the tissues containing undifferentiated cells or partially differentiated cells (Fig. 5d). These results support our assumption that relatively high Taz expression is one of the critical causes of the differentiation defects observed upon Mob1a/b depletion.

Taz expression was significantly increased in Mob1a/b-depleted ESCs upon differentiation (Supplementary Fig. 3). To investigate the role of Taz and Yap in the differentiation of mouse ESCs, we knocked down Taz or Taz/Yap expression using shRNA gene silencing in Mob1a/b-depleted ESCs (Supplementary Fig. 5). Downregulation of Taz or Taz/Yap in mouse ESCs had little effect on *Oct4* expression, alkaline phosphatase activity, or colony morphology in the undifferentiated state. However, downregulation of these two proteins individually or together in Mob1a/b-depleted ESCs overcame the in vitro differentiation defects, at least in part, caused by Mob1a/b depletion. These results were similar to those obtained with shYap ESCs and suggest that Taz and Yap have a redundant function in the differentiation of mouse ESCs into the three germ layers.

Taken together, these results suggest that Mob1a/b–Yap signaling (i.e., the Hippo signaling pathway) plays a critical role in the formation of differentiated tissues or cells from ESCs as well as the specification of the three germ layers.

## Discussion

Because Mob1a/b is a negative regulator of Yap^32^ and the pluripotency of ESCs requires hyperactive Yap^20^, we hypothesized that Mob1a/b depletion would cause little change in ESCs. As expected, the self-renewal and pluripotency of mESCs were well maintained despite complete depletion of Mob1a/b. In contrast, Yap must be suppressed during the differentiation of mouse ESCs. Indeed, Yap overexpression prevents ESC differentiation^20^. Thus, Mob1a/b is likely to play an essential role in differentiation as a negative regulator of Yap.

Whereas Lian et al.^20^ reported that the protein level of Yap was significantly decreased in the differentiation condition and Yap knockdown resulted in the loss of ESC characteristics, we observed that Yap-S112 phosphorylation was increased in the differentiation condition with little change in the protein level of Yap (Supplementary Fig. 3). Moreover, Yap knockdown had little effect on colony formation, alkaline phosphatase staining, or Oct4 level in mouse ESCs (Fig. 4a and Supplementary Fig. 5). Different studies have used different mouse ESC lines and feeder cells. Whereas Lian et al.^20^ used ESC line and feeder cells derived from the 129/Sv strain, we established an ESC line from C57BL/6 blastocysts and used C57BL/6-derived MEF cells. We suggest that the use of mouse ESC lines and feeder cells derived from different mouse strains could result in these conflicting results of the two groups^47–49^.

In this study, depletion of Mob1a/b prevented the differentiation of mouse ESCs into the three germ layers. In the process of the differentiation of Mob1a/b-depleted ESCs, the levels of stem cell and germ layer markers did not change and were similar to those in undifferentiated wild-type ESCs (Figs. 2c and 3a). These results are different from a previous report, which showed that only primitive endoderm markers were significantly suppressed in Mob1a/b-depleted EBs^30^. In the time-dependent EB formation assays of our study, each differentiation marker was upregulated at different differentiation stages or days. For example, *T-brachyury* expression was the highest on day 4 after EB formation, but *Hand1* expression was the highest at day 6 (Fig. 3c). These results suggest that the differences between wild-type and Mob1a/b-deficient EBs are dependent on which differentiation stages are set as the criteria.

In the teratoma formation assay, teratomas generated with Mob1a/b-depleted ESCs did not show any structural characteristics of the three germ layer cells (Fig. 5a). In addition, Yap knockdown in the Mob1a/b-depleted ESCs showed only partial restoration of differentiation into the three germ layers compared to the control (wild-type or Yap-knockdown ESCs) (Fig. 5a, b). These results indicate that Yap downregulation together with the up- or downregulation of an additional factor(s) is required to rescue the in vivo defects in differentiation observed in Mob1a/b-depleted ESCs. Based on our observation of elevated Taz levels in Mob1a/b-depleted teratomas, downregulation of both Yap and Taz could restore the normal differentiation of mouse ESCs into the three germ layers in vivo.

Recent studies have shown that activation of Yap is sufficient to change differentiated cells to stem or progenitor cells in adult organs and tissues, especially airway epithelium and liver^50,51^. Yap activity was also required for the regeneration of adult tissues following injury^44,52–55^. These results suggest that therapeutic suppression of Hippo signaling or elevation of Yap activity can therapeutically improve the efficiency of tissue regeneration and repair upon injury. However, pharmacological manipulation of Yap activity for practical application in regenerative medicine must be developed to transiently activate Yap and reduce the detrimental side effects by its activation because Yap is known as an oncogenic protein, and failure to suppress its activity has been reported in a broad range of human cancers^56^. Because Mob1a/b is the core component of the Hippo pathway, targeting Mob1a/b as well as other Hippo signaling components may be beneficial in developing the pharmacological manipulations of Yap activity in regenerative medicine.

In summary, we found that depletion of Mob1a/b, the core component of the Hippo pathway, caused a defect in the differentiation of mouse ESCs into the three germ layer lineages, which is dependent on Yap. These results suggest a close relationship between the Hippo pathway and the differentiation of stem cells and its potential as a therapeutic target for tissue regeneration and repair.

## Supplementary information

Supplementary information
