# Supplementary material for: Loss of Mob1a/b impairs the differentiation of mouse embryonic stem cells into the three germ layer lineages
Source: Exp Mol Med. 2019 Nov 14;51(11):135. doi: 10.1038/s12276-019-0342-z (PMC6853965; doi:10.1038/s12276-019-0342-z)
Supplement: Supplementary file 1 — Supplementary information [file 12276_2019_342_MOESM1_ESM.pdf]

Supplementary information

Loss of Mob1a/b impairs the differentiation of mouse embryonic stem cells into the three germ layer lineages.

June Sung Bae[1], Sun Mi Kim[2], Yoon Jeon[1], Juyeon Sim[1], Ji Yun Jang[1], Jaehyung Son[2], Woosol Hong[2], Mi Kyung Park[2] and Ho Lee[1,2]

[1] Research Institute, [2] Graduate School of Cancer Science and Policy, National Cancer Center, Goyang, Gyeonggi 10408, Republic of Korea

Correspondence to:

Ho Lee, PhD. Graduate School of Cancer Science and Policy, National Cancer Center, Gyeonggi 10408, Republic of Korea, Phone +82-31-920-2274, Fax +82-31-920-2279, E-mail:ho25lee@ncc.re.kr

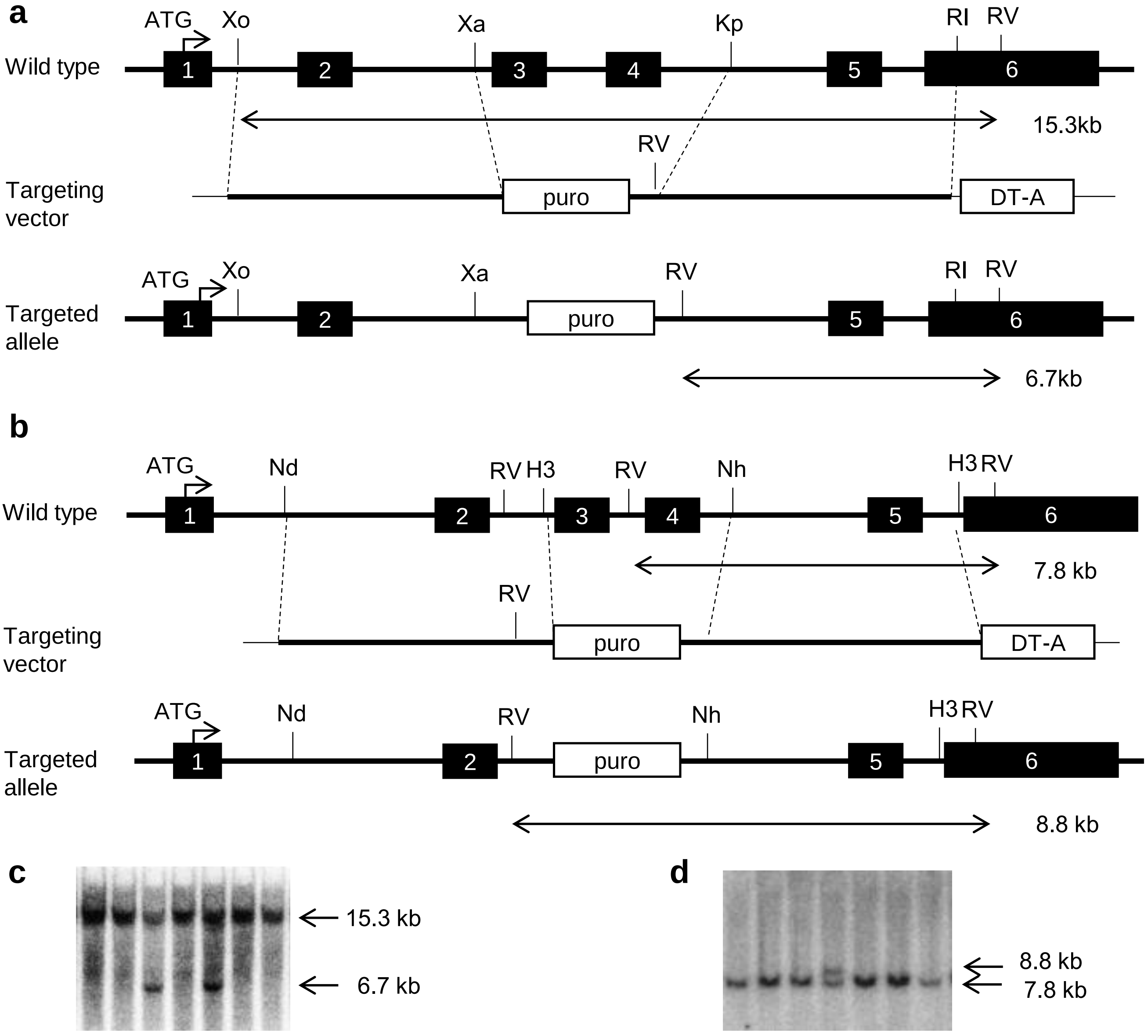

Supplementary Figure 1. Targeting of the *Mob1a* and *Mob1b* loci. **a, b** Schematic representation of the *Mob1a* and *Mob1b* loci, the targeting vector, and the targeted loci are shown. Exon 1~6, translation start site (ATG), puromycin cassette (puro), diphtheria toxin A chain gene (DT-A), and restriction sites (Xo, XhoI; Xa, XbaI; Kp, KpnI; RI, EcoRI; RV, EcoRV; Nd, NdeI; H3, HindIII; Nh, NheI) are shown. **c** Southern blot analysis of a representative *Mob1a*$^{+/puro}$ ES cell clones is shown. For XhoI/EcoRV digestion, the bands representing wild-type and mutant alleles are ~15.3 kb and ~6.7 kb, respectively. **d** Southern blot analysis of a representative *Mob1b*$^{+/puro}$ ES cell clones is shown. For EcoRV digestion, the bands representing wild-type and mutant alleles are ~7.8 kb and ~8.8 kb, respectively.

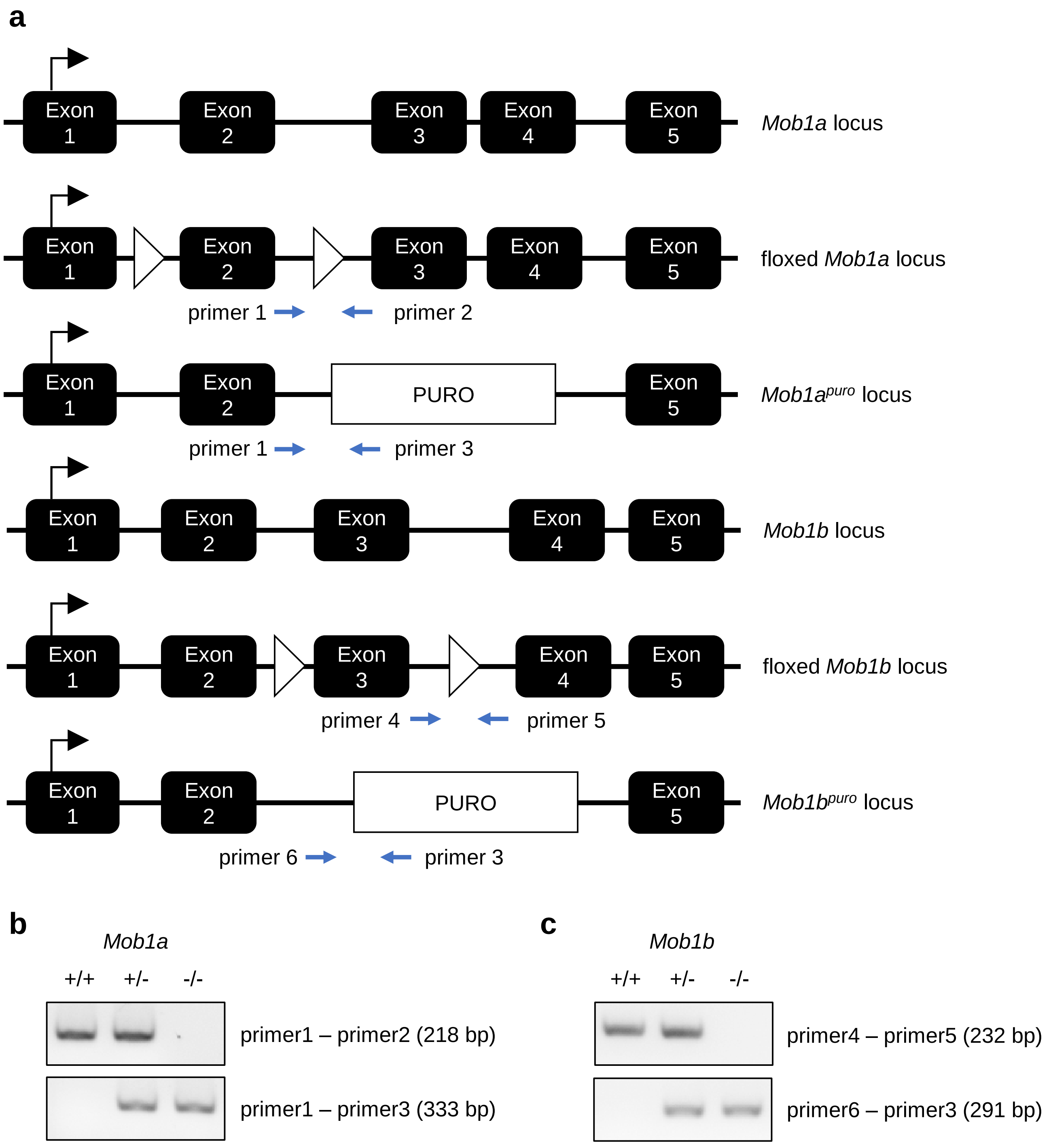

Supplementary Figure 2. Generation of *Mob1a/b* knockout mice. **a** Schematic diagram of generation of *Mob1a/b* conventional and conditional knockout mice. **b, c** Genotyping of *Mob1a^{puro}* and *Mob1b^{puro}* alleles.

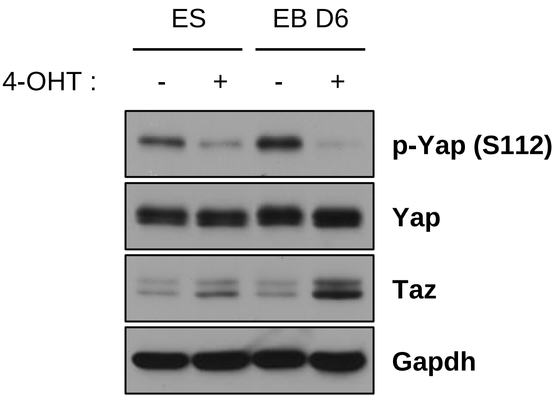

Supplementary Figure 3. Immunoblot analysis for phospho-Yap (S112), Yap and Taz in lysates from *Mob1a*$^{f/-}$: *Mob1b*$^{f/f}$: *CAGGCre-ER*^{TM} mouse ESCs and EBs. Gapdh served as a loading control. ES, undifferentiated ESCs; EB D6, EB after 6-day culture (in cell and tissue culture dish).

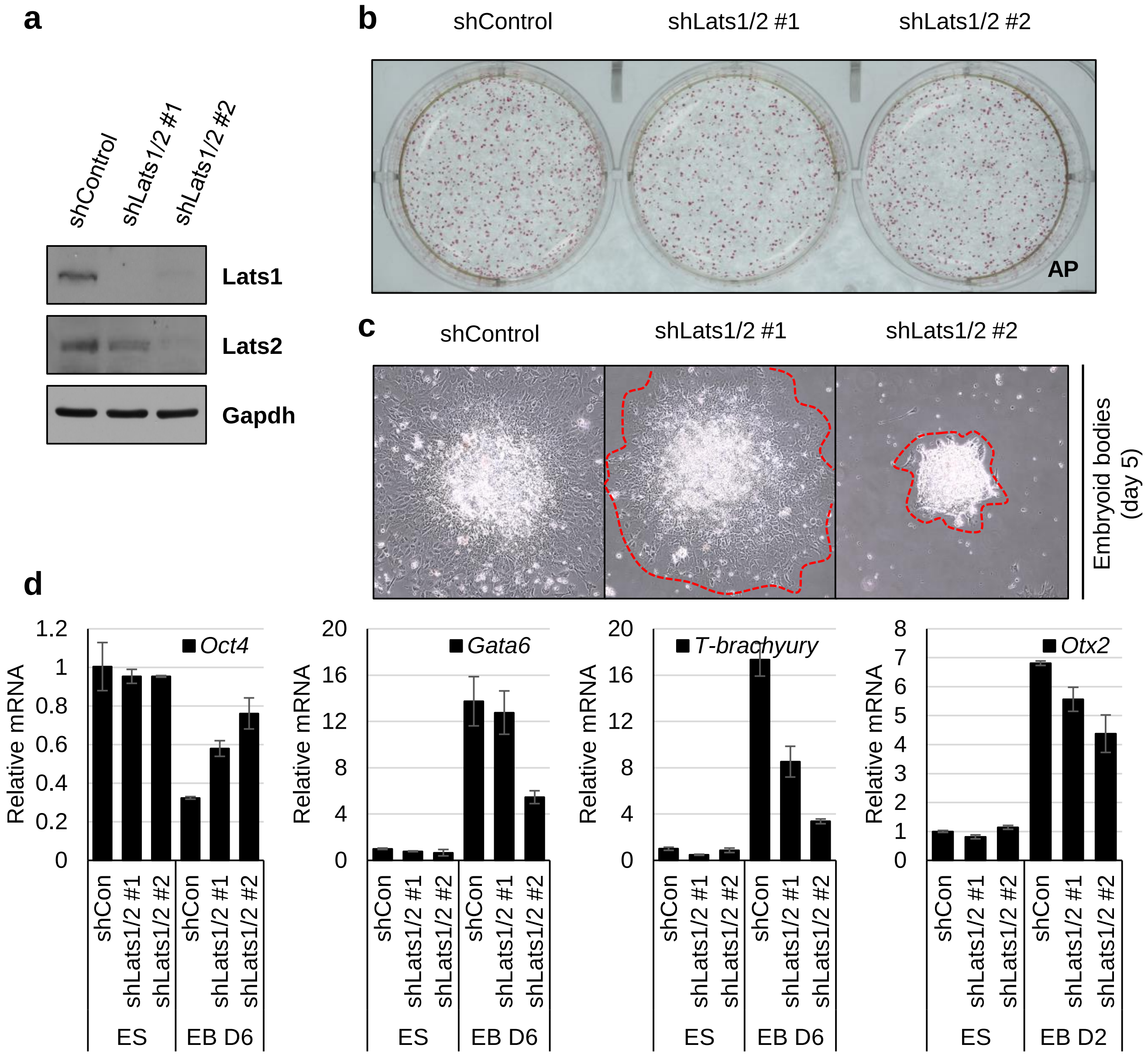

Supplementary Figure 4. a Immunoblot analysis for Lats1 and Lats2 in lysates from shLats1/2 #1 (#2 target sequence of Lats1 and #2 target sequence of Lats2) and #2 (#3 target sequence of Lats1 and #3 target sequence of Lats2) mouse ESCs described in Materials and Methods. **b** Colony formation and alkaline phosphatase staining of shLats1/2 ESCs. **c** Representative images of EB outgrowth on cell/tissue culture dishes on the day 5 after EB formation. **d** RT-qPCR for *Oct4*, *Gata6*, *T-brachyury*6, and *Otx2* in mESCs and EBs. ES, undifferentiated ESCs; EB D2, EBs after 2-day culture (hanging drop stage); EB D6, EBs after 6-day culture (in cell/tissue culture dish).

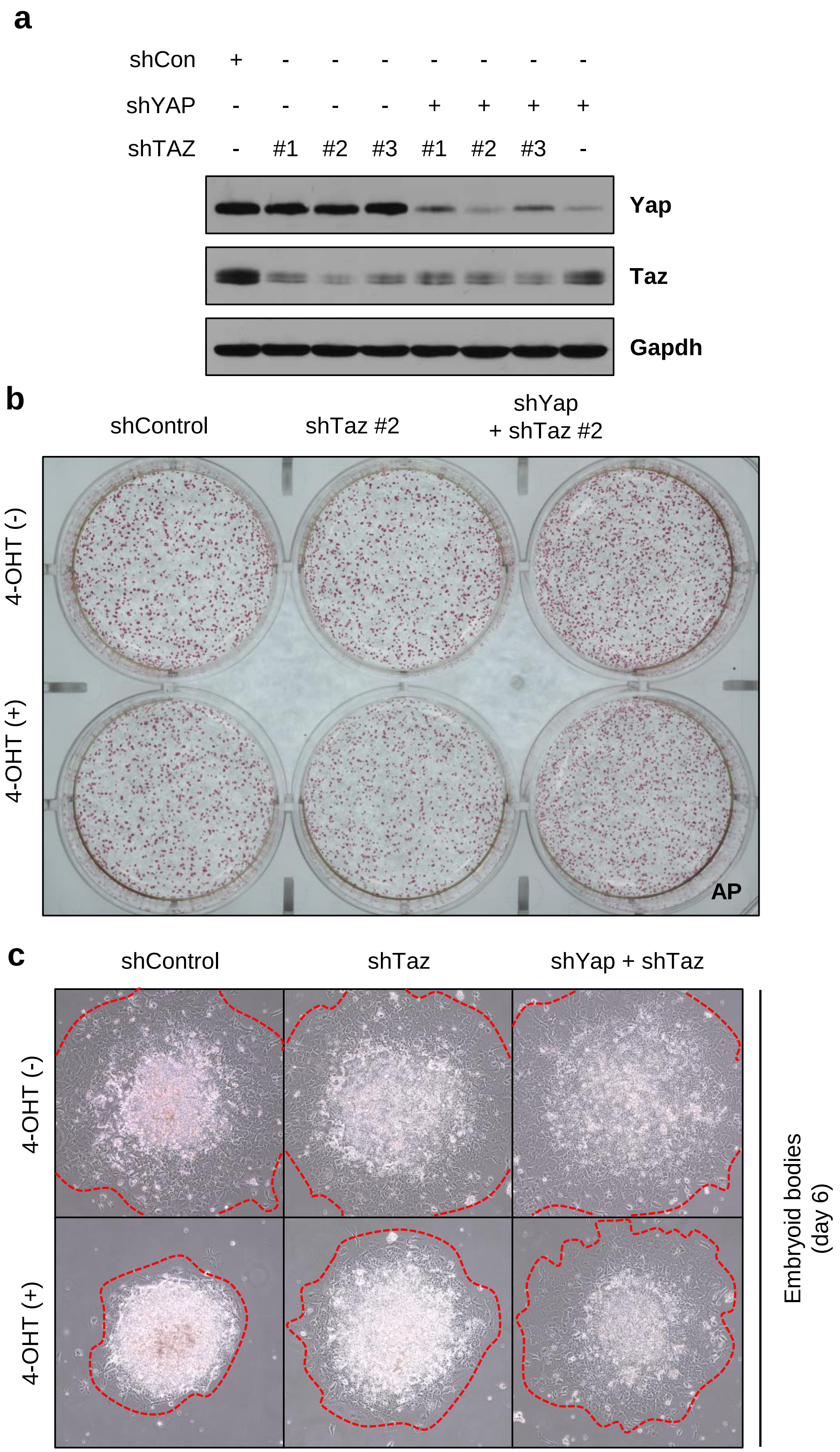
a
shCon + - - - - - - -
shYAP - - - - + + + +
shTAZ - #1 #2 #3 #1 #2 #3 -
Yap
Taz
Gapdh
b
shControl
shTaz #2
shYap
+ shTaz #2
4-OHT (-)
4-OHT (+)
AP
c
shControl
shTaz
shYap + shTaz
4-OHT (-)
4-OHT (+)
Embryoid bodies
(day 6)

d

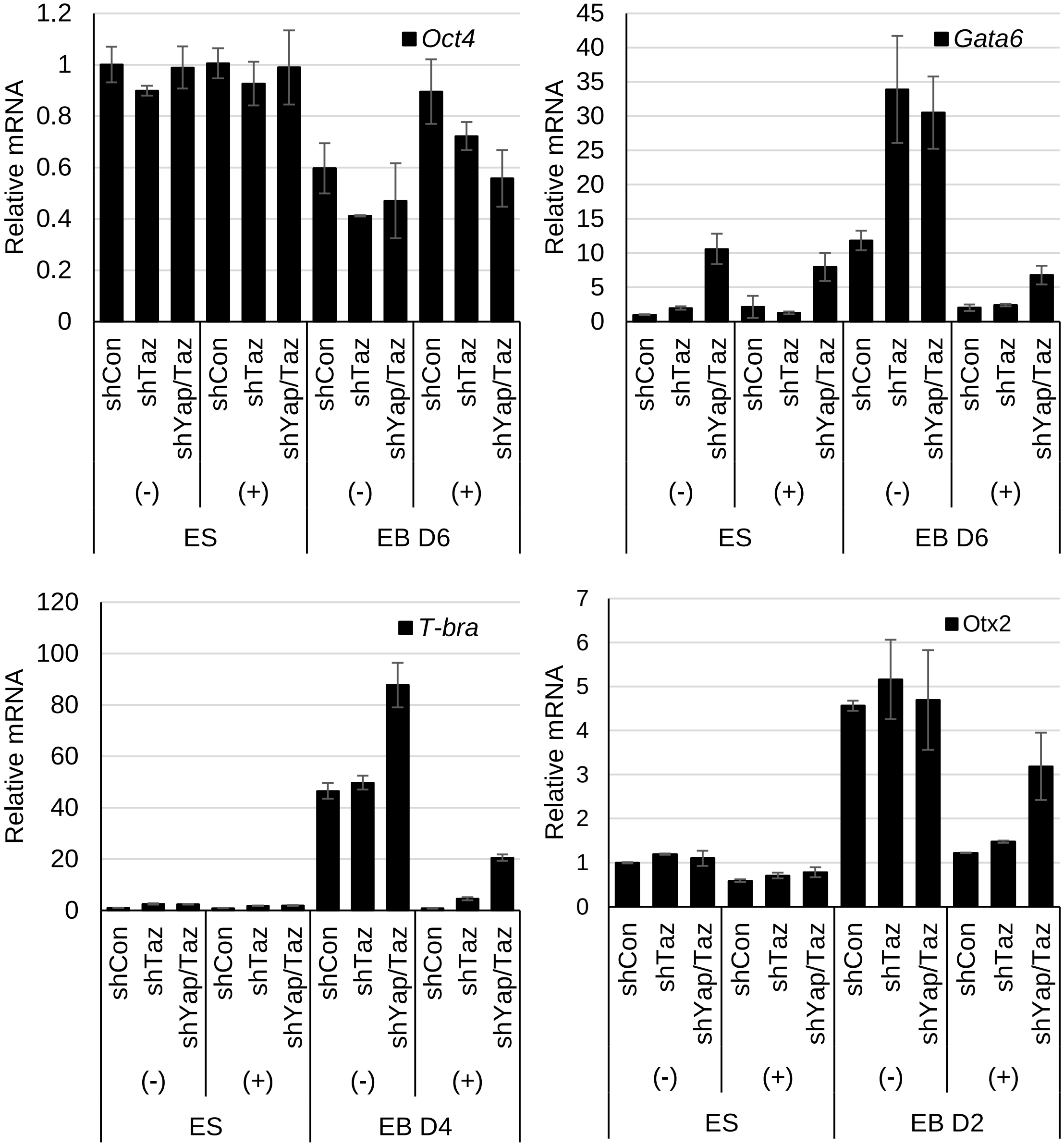

Supplementary Figure 5. a Immunoblot analysis for Yap and Taz in lysates from *Mob1a*$^{f/f}$: *Mob1b*$^{f/f}$: *CAGGCre-ER*^{TM} mouse ESCs. **b** Colony formation and alkaline phosphatase staining of shTaz and shYap/Taz ESCs. **c** Representative images of EB outgrowth on tissue culture dishes on the day 5 after EB formation. **d** RT-qPCR for *Oct4*, *Gata6*, *T-brachyury*, and *Otx2* in mESCs and EBs. ES, undifferentiated ESCs; EB D2, EBs after 2-day culture (hanging drop stage); EB D4, EBs after 4-day culture (in bacteriological grade Petri dish); EB D6, EBs after 6-day culture (in cell/tissue culture dish).

Antibody	Manufacturer	Order number
Rabbit polyclonal against Mob1a/b	Cell signaling	#3863
Mouse monoclonal against Oct4	Santa Cruz Biotechnology	sc-5279
Goat polyclonal against Nanog	Santa Cruz Biotechnology	sc-30328
Rabbit polyclonal against YAP	Cell signaling	#4912
Rabbit polyclonal against phopho-YAP (S127)	Cell signaling	#4911
Rabbit polyclonal against TAZ	Sigma	HPA007415
Rabbit polyclonal against Lats1	Abcam	ab70561
Rabbit polyclonal against Lats2	Abcam	ab135794
Mouse polyclonal against GAPDH	Santa Cruz Biotechnology	sc-32233
Goat anti-mouse IgG conjugated HRP	GenDEPOT	SA001-500
Goat anti-rabbit IgG conjugated HRP	GenDEPOT	SA002-500
Rabbit anti-goat IgG conjugated HRP	GenDEPOT	SA007-500

Supplementary Table 1. Primary antibodies for immunoblotting and immunohistochemistry.

Gene	Forward primer (5′ to 3′)	Reverse primer (5′ to 3′)
Mob1a	TCG GAA GTG GCA ATC TGA GG	CAG CTT GCC TCA GTG CAG AA
Mob1b	CTA CGG ATG GCT GTC ATG CT	GAC ATC ACC GGA CAG CTC TC
Oct4	TTG GGC TAG AGA AGG ATG TGG TT	GGA AAA GGG ACT GAG TAG AGT GTG G
Sox2	GCA CAT GAA CGG CTG GAG CAA CG	TGC TGC GAG TAG GAC ATG CTG TAG G
Nanog	AAC CTT TTC AGA AAT CCC TTC C	GAG GCA GGT CTT CAG AGG AA
Gata6	GCT GAA CGG AAC GTA CCA CC	ACA GTG GCG TCT GGA TGG AG
Gata4	CCT GGA AGA CAC CCC AAT CTC	AGG TAG TGT CCC GTC CCA TCT
Sox17	AAA GCG GAG TCT CGC ATC C	CGC TTC TCT GCC AAG GTC AA
FoxA1	CAA GGA TGC CTC TCC ACA CTT	TGA CCA TGA TGG CTC TCT GAA
Pdgfra	TCC ATG CTA GAC TCA GAA GTC A	TCC CGG TGG ACA CAA TTT TTC
Hand1	TCG CCT ACT TGA TGG ACG TG	GGC CTG GTC TCA CTG GTT TA
T-brachyury	CAC CGC TGG AAA TAT GTG AA	CAC GAT GTG AAT CCG AGG TT
Twist2	CAG TGA GGA AGA GCT GGA GAG G	CTG GAT CTT GCT GAG CTT GTC A
FoxA2	AGC ACC ATT ACG CCT TCA AC	CCT TGA GGT CCA TTT TGT GG
MixL1	CGT CTT CCG ACA GAC CAT GT	GTT CTG GAA CCA CAC CTG GAT
Fgf5	GCC TGT CCT TGC TCT TCC TCA T	GGA GAA GCT GCG ACT GGT GA
Otx2	GCG AAG GGA GAG GAC GAC ATT T	CTG CTG TTG GCG GCA CTT AG
Sox1	GCC GAG TGG AAG GTC ATG TC	TTG AGC AGC GTC TTG GTC TTG
Pax6	ACC AGT GTC TAC CAG CCA ATC C	GCA CGA GTA TGA GGA GGT CTG A
Eomes	CCA CCG CCC ACT ACA ATG TT	CCC AAA GGA AAT CTC CTG CCT
Nestin	GCT GGA ACA GAG ATT GGA AGG	CCA GGA TCT GAG CGA TCT GAC
Gapdh	TGT CGT GGA GTC TAC TGG TGT C	GCT AAG CAG TTG GTG GTG CAG G

Supplementary Table 2. Primer sequences used for quantitative or semiquantitative PCR.

	Sequences (5′ to 3′)
Primer 1	CCTCGTCGTCTGGATCTAGC
Primer 2	CCAGCATACTGAAGAC CACTC
Primer 3	GCTACTTCCATTTGTCACGTCCTGCACGA
Primer 4	CCATCTCTTCAGCCTCCCTTCTTG
Primer 5	GAGCTTAGTGCTAGAGAGATGAC
Primer 6	ATGTCTTCTTGCTCTCTAA TAC

Supplementary Table 3. Primer sequences for genotyping of *Mob1a/b* alleles shown in Supplementary Figure 2.
